# Intragenomic Matching Reveals a Huge Potential for miRNA-Mediated Regulation in Plants

**DOI:** 10.1371/journal.pcbi.0030238

**Published:** 2007-11-30

**Authors:** Morten Lindow, Anders Jacobsen, Sanne Nygaard, Yuan Mang, Anders Krogh

**Affiliations:** Bioinformatics Centre, Department of Molecular Biology and Biotech Research and Innovation Centre, University of Copenhagen, Copenhagen, Denmark; Ceres Inc., United States of America

## Abstract

microRNAs (miRNAs) are important post-transcriptional regulators, but the extent of this regulation is uncertain, both with regard to the number of miRNA genes and their targets. Using an algorithm based on intragenomic matching of potential miRNAs and their targets coupled with support vector machine classification of miRNA precursors, we explore the potential for regulation by miRNAs in three plant genomes: *Arabidopsis thaliana, Populus trichocarpa,* and *Oryza sativa.* We find that the intragenomic matching in conjunction with a supervised learning approach contains enough information to allow reliable computational prediction of miRNA candidates without requiring conservation across species. Using this method, we identify ∼1,200, ∼2,500, and ∼2,100 miRNA candidate genes capable of extensive base-pairing to potential target mRNAs in *A. thaliana, P. trichocarpa,* and *O. sativa,* respectively. This is more than five times the number of currently annotated miRNAs in the plants. Many of these candidates are derived from repeat regions, yet they seem to contain the features necessary for correct processing by the miRNA machinery. Conservation analysis indicates that only a few of the candidates are conserved between the species. We conclude that there is a large potential for miRNA-mediated regulatory interactions encoded in the genomes of the investigated plants. We hypothesize that some of these interactions may be realized under special environmental conditions, while others can readily be recruited when organisms diverge and adapt to new niches.

## Author Summary

microRNAs (miRNAs) are small RNA molecules that regulate gene expression by complementary basepairing to mRNAs. In plants, this base-pairing is almost perfect along the whole length of miRNAs. This long stretch of complementarity makes it relatively easy to make computational predictions of the targets for known miRNAs. To predict novel miRNA genes, we take advantage of this and reverse the target prediction: instead of predicting targets for known miRNAs, we predict novel miRNA candidates for all known mRNAs. Because matching between target and miRNA candidates is integral to the method, it is possible to achieve good predictions without having to rely on evolutionary conservation, as most other current methods do. This means that we can predict new miRNAs that are specific to an organism. Interestingly, this could help explain the difference between species that have very similar protein-coding genes, but highly different phenotypes. Furthermore, it turns out that many of these new miRNA candidates derive from genomic repeat regions such as transposons, which points to a possible active role for repeats/transposons in the regulation of gene expression.

## Introduction

Small RNAs are now accepted as major players in the control of eukaryotic gene expression. Most well known are microRNAs (miRNAs) and small interfering RNAs (siRNAs), both of which are derived from the processing of dsRNA molecules by members of the Drosha/Dicer family of endonucleases. In plants, siRNA and miRNA are distinguished mainly by their biogenesis, not by their mechanism of action. MiRNAs arise from stem-loop precursors encoded in the genome, and their major mechanism of action in plants is thought to be post-transcriptional regulation through near-complementary base-pairing to target mRNAs, leading to specific endonucleolytic cleavage and degradation of the target [1].

Most of the initially discovered miRNAs were so highly conserved in evolution that a defining characteristic of a miRNA was that it *had to be conserved* [2]. This attribute of those miRNAs discovered early has been used successfully by a number of groups to computationally predict new miRNA genes [3–6]. Basically, these methods scan the genome for inverted repeats with the potential to form miRNA precursors. Such scans typically find on the order of hundreds of thousands to millions of hairpins, depending on genome size and search parameters [4] (plus our own unpublished data). This high number is then reduced by only keeping hairpins that are conserved in other species. Another approach is to search only transcribed sequences in the form of expressed sequence tags [7,8]. This method works for nonsequenced genomes and efficiently reduces the search space, probably leading to a lower number of false positives, but the method also misses candidates not covered by the expressed sequence tag libraries.

In miRBase version 8.2, Arabidopsis thaliana (*Arabidopsis*) has 118 miRNA genes listed, most of which are conserved down to the monocot Oryza sativa (*Oryza*)*.* However, studies of noncoding RNA have shown that lack of conservation does not necessarily mean lack of function [9]. Potentially, all it takes to evolve a miRNA is for one of the many inverted repeats in the genome to be transcribed and have the necessary structure and sequence features to be recognized and processed by Drosha/Dicer. Indeed, large numbers of more narrowly conserved miRNAs also exist [10]. A recent bioinformatic study in human identified patterns associated with miRNA precursors and suggested that the number of miRNA precursors is larger than 25,000 [11]. In plants, a similar situation could exist. A deep sequencing effort in *Arabidopsis* using the massively parallel signature sequence (MPSS) technique has revealed 75,000 distinct small RNA species (not all miRNAs, though) [12] mapping to a large variety of genomic contexts, including exons, introns, repetitive DNA, and intergenic regions. This is perhaps not surprising considering other studies finding that unexpectedly large fractions of eukaryotic genomes are transcribed also outside and antisense to annotated protein-coding genes [13–15].

A necessary feature of any functional miRNA is that it must target at least one mRNA. In plants, this means that the miRNA must be almost complementary to some part of the spliced mRNA transcript (not just the 3′ untranslated region as is currently thought to be the main target for animal miRNAs). A set of rules allowing mismatches only in certain positions has been suggested based on experimental observations [16]. The requirement for a target has previously been used to predict plant miRNAs [17–19]: instead of (or in addition to) relying on phylogenetic conservation (*intergenomic* matches), these methods have successfully used *intragenomic* matches with potential target mRNAs to find the hairpins potentially capable of producing miRNAs that can regulate the target(s). Such intragenomic matches will inherently arise from the structure and dynamics of the genome: retrotransposons, formation of pseudogenes, and other duplicative events provide sequences almost ready to regulate the originally copied gene [20]; likewise, the reverse strand of one gene is complementary to other paralogous genes. By not relying on conservation between species, intragenomic matching is capable of more fully charting the potential for post-transcriptional regulation by miRNAs.

In an effort to reduce spurious predictions, earlier screens for new miRNAs have removed candidates overlapping existing annotation, such as repeats and protein-coding regions. Although such filters probably increase the signal-to-noise ratio, they also introduce biases assuming that repeat-derived sequences are not functional and that each sequence segment can have only one function. However, transposon-derived conventional miRNAs have been demonstrated in *Arabidopsis* [21], and recent work of several groups show that repeat-associated miRNAs are quite common in mammals [22–26]. Borchert et al. point to 50 human miRNAs that are associated with Alu repeats and polymerase III transcription [22]. Piriyapongsa et al. link 55 experimentally characterized human miRNAs to different types of transposable elements [26]. Of these, 18 are conserved in other vertebrate genomes, and the authors predict an additional 85 novel transposable element–derived miRNAs. These observations, along with the evidence of very complex and widespread transcriptional patterns in eukaryotes, including nested transcripts and antisense transcription [27], underlines the importance of enumerating all possible miRNA/target interactions in order to explore the full potential of miRNA-mediated regulation.

In this paper, we develop and apply the miMatcher pipeline to perform intragenomic matching followed by classification of miRNA candidates using support vector machines (SVMs). Using this method in the three plant genomes A. thaliana, *O. sativa,* and *P. trichocarpa,* we find species-specific miRNA-like hairpins (miRNA candidates) with almost perfect complementarity to mRNA targets. We present indications that many of these are active and hypothesize that the remainder forms a pool of regulators, which can easily be recruited by natural selection on the adapting organisms.

## Results/Discussion

### miMatcher Pipeline: Prediction of miRNA Genes and Targets Using Intragenomic Matching and an SVM

The computational procedure builds on our previously published method [18] that predicted potential miRNA genes in *Arabidopsis,* most of which are not conserved in *Oryza*. Three of these previous predictions (all nonconserved) have subsequently been confirmed as being expressed and correctly processed into small RNAs (T. Dezulian, personal communication, unpublished data).

**Figure 1:**
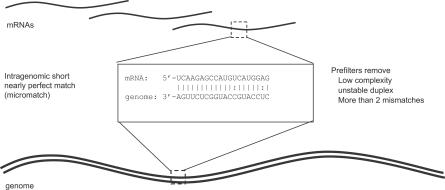
Conceptual Model of Intragenomic Matching mRNA sequences are matched against the genome and matches are prefiltered. Matches with miRNA precursor potential are selected for further processing.

**Figure 2:**
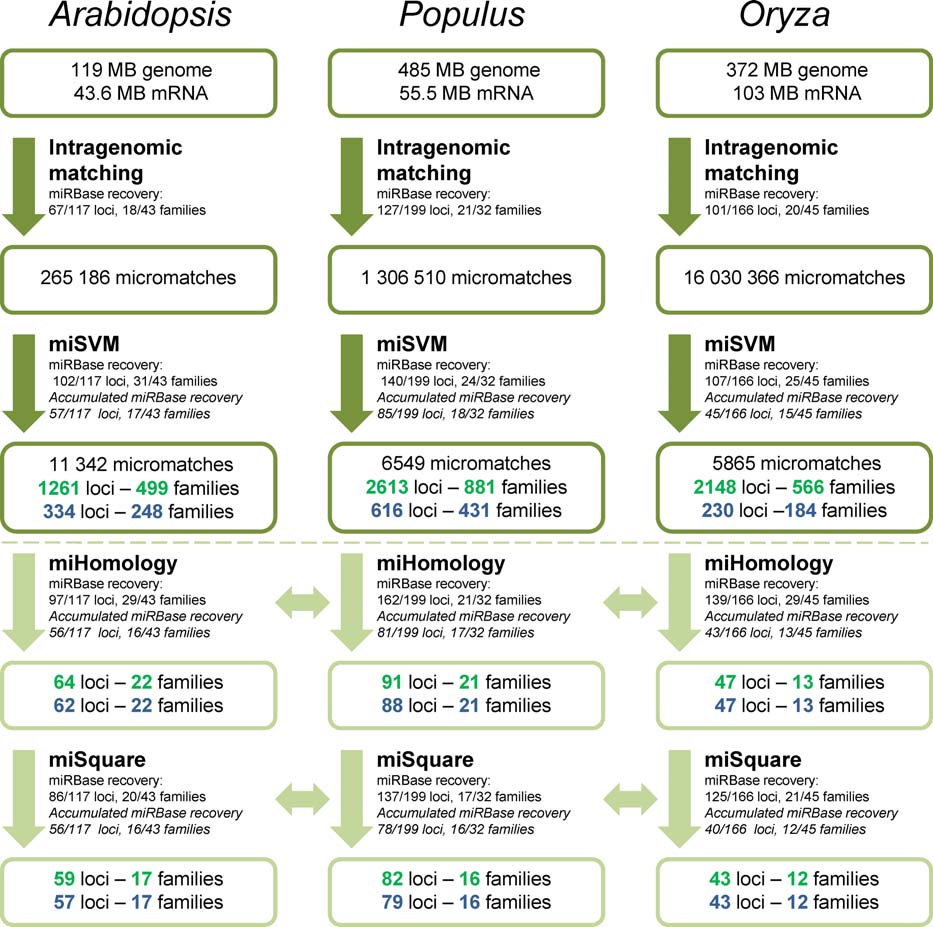
Overview of the Number of miRNA Candidates at Successive Steps of the Procedure A genome assembly and a set of annotated mRNA transcripts are input to the intragenomic matching. *Intragenomic matching.* The result of the intragenomic matching generates “micromatches” consisting of pairs of a genome segment and an mRNA segment. Also shown is the recovery of miRBase 8.2 loci and families. *miSVM.* Remaining number of miRNA loci and families after miSVM classification is shown (numbers in green). The number of miRNA candidate loci and families not overlapping repeat/CDS regions are shown in blue. *miHomology.* Conservation filters were applied to detect the subset of miRNA candidates that have at least one homolog in one of the other two organisms. *miSquare.* The conserved miRNA candidates with the additional requirement of targets orthologs.

The miMatcher procedure predicts miRNA candidates and their targets independently in each plant genome. First, we enumerate all intragenomic matches between any mRNA and any other part of the genome, where the genomic part of the match is able to bind complementarily to the mRNA part of the match (Figure 1). We call such a match a “micromatch.” The assumption is that the genomic part can be a miRNA gene that targets the mRNA. Looking at micromatches between known *Arabidopsis* miRNAs and their targets, we have derived a set of rules that the match must fulfill: we start from the observation that targets can be found above noise without using phylogenetic conservation by requiring no more than two mismatches [19]. The match length is required to be between 20 and 25 nucleotides, and we add previously described filters for low complexity [5] and low-binding free energy [18]. Furthermore, for a genomic match to be a potential miRNA gene, it must be part of a sequence that can fold into a stem-loop precursor recognizable by the biosynthetic machinery that makes miRNAs. A necessary (but not sufficient) requirement for this is that the match (potential mature miRNA) must form base pairs in one direction only; i.e., the mature miRNA forms base pairs with bases either upstream or downstream. Figure 2 (step 1) shows the number of candidate matches that pass these prefilters for each organism (see Materials and Methods for a detailed explanation of the filters).

**Figure 3:**
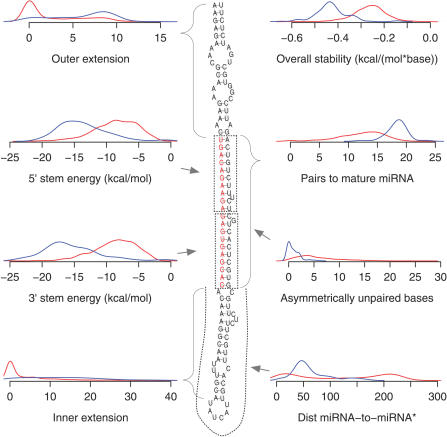
The Structural Feature Space of miSVM Distribution of structural features in the positive (blue) and negative (red) examples used to train miSVM. Arrows illustrate the feature on an example miRNA precursor, with the mature miRNA sequence highlighted in red.

Not all stem-loop structures can work as dicer substrates. To distinguish those that do work from those that do not, we analyze a range of structural attributes for each candidate. Figure 3 illustrates the structural attributes that we investigated (see Materials and Methods for details).

Next, we build a classifier capable of selecting the stem-loops (having at least one target) most likely to be true miRNA genes based on the attributes summarized in Figure 3. To this end, we construct a positive and a negative control set. While the positive controls are simply the known miRBase miRNAs for each plant (regardless of whether we can find a target for them or not), the negative control set is less obvious to construct: we rely on the assumption that all miRNAs that regulate a known target as identified by [28] is already known. Accepting that this assumption is fairly reasonable means that that we can generate a negative control set by running the intragenomic matching (including prefilters as above) with the “known targets” as queries and then removing those genomic matches that overlap with already known miRBase miRNAs. Then, for each place in the genome matching a query mRNA, the flanks are extracted and the minimum free energy structure is calculated. The minimum free energy structure is analyzed, and structural features are calculated.

**Figure 4:**
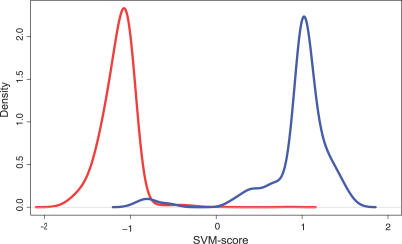
Performance of miSVM Density of the miSVM score of positive (blue) and negative examples (red).

For most of the measures, there is a clear separation between the positive and negative control sets (Figure 3; red and blue traces, respectively), but there are still unnegligible overlaps. This shows that if we filter by hard threshold values on each attribute, we will either lose a large portion of the true positives or be forced to allow a large number of false positives to pass through the filters. Instead, we use an SVM [29] to classify based on all the attributes to achieve maximum separation. SVMs have successfully been used for animal miRNA precursor structure classification [30], but not yet for plants. We train an SVM individually on each species, which is important because some of the input are values for the RNA folding and hybridization, which is strongly influenced by the GC composition of the genomes. Figure 4 shows separation of the miSVM score between positive and negative examples, and Table 1 lists performance estimates using cross-validation (see Materials and Methods). In *Arabidopsis*, according to the cross-validation, when searching for miRNA candidates targeting a specific mRNA, 93.7% of all the positively classified candidates returned (if any) will be true positives. This specificity, however, comes at a price: 27% of the *Arabidopsis* miRBase miRNAs are erroneously classified as non-miRNAs. This remarkably specific identification of the known miRNA genes shows that intragenomic matching according to a strict set of targeting rules followed by classification on the basis of structural features of the precursor is sufficient for prediction of novel miRNA candidates. In the other species, the performance is comparable, albeit slightly less specific.

**Table 1:**
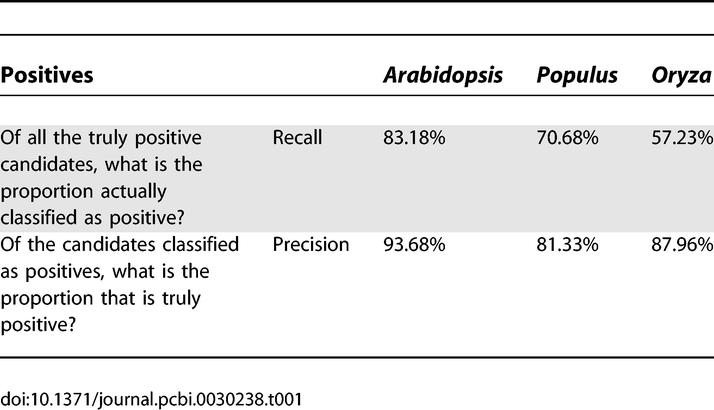
Performance of miSVM Estimated from Cross-Validation

In contrast to other methods, our method does not depend on conservation in other genomes, and is therefore able to predict species-specific miRNA candidates.

A summary of the results of applying miMatcher followed by miSVM to three plant genomes is shown in Figure 2. After classification, the positively classified micromatches are grouped into *candidate loci* on the basis of the genomic positions and *families* according to miRNA sequence similarity (see Materials and Methods). We find 1,261, 2,613, and 2,148 candidate miRNA loci in *Arabidopsis, Populus,* and *Oryza,* respectively (Datasets S1–S3). The fact that these different genomes despite their genome sizes and structures (i.e., *Oryza's* peculiar repeat genome structure [31]) have around the same number of candidate miRNAs with targets is striking and supportive of the method.

When comparing the classification by miSVM with a recently suggested rule-based classification of *Arabidopsis* pre-miRNAs [32], miSVM is much more stringent: the rules-based method accepts 100 out of 107 in our positive examples (compared to 82 of 107 for miSVM), but it fails to reject 224 of 1,372 of the negative examples.

A recent review [32] questioned some miRBase-registered miRNAs (ath-MIR413 to 420 and ath-MIR426) found in a study relying on miRNA conservation between *Arabidopsis* and *Oryza* [6]. These miRNAs seem to lack conservation in organisms outside *Arabidopsis* and *Oryza*, and when tested, they gave weak hybridization signals on Northern blots. Moreover, they have less pairing in the miRNA precursor stem than many of the other miRBase miRNAs. Interestingly, these nine miRNAs are not among the predicted miRNAs coming through the miMatcher pipeline steps, and six of them (ath-MIR413 and ath-MIR417 to 426) are among the 25 “false negatives” we get in the above miSVM evaluation.

### Candidates in Repeat Regions

We use two methods to classify candidates as derived from repetitive regions: (1) RepeatMasker to find known repeats and transposable elements as well as simple low-complexity sequences; but since this relies on the quality of the available repeat libraries, we also (2) count the copy number of the mature candidate miRNA sequence in the whole genome, regarding candidates with high copy numbers (>100) as repetitive (see Materials and Methods). Following this classification, we find that although underrepresented, there is still a sizeable fraction of the known miRBase miRNA mapping to repeats (8%–16%).

However, since most miRBase miRNAs are located outside repeat and coding regions, we investigated the effect of removing such candidates and found that it reduces the number of candidate miRNAs significantly (Figure 2). Only about one-fourth of the candidates remain in *Arabidopsis* and *Populus*, and in *Oryza,* the number is reduced to around 10%. While it might be argued that the risk of false positives in the repeat and coding regions is higher, it is striking that there is a very large potential for miRNAs in such regions, and we speculate that the lack of experimental evidence could in part be due to them being actively excluded in previous studies.

Because candidates encoded in repetitive or protein-coding segments (CDS) of the genome could be qualitatively different from those derived from other regions, we have chosen to focus on the nonrepeat/non-CDS candidates in the following analyses.

### Conservation Analysis

While conservation is not a requirement for our miRNA candidates, knowing whether a candidate has homologs in other species is useful and does strengthen the reliability of the prediction. To explore the conservation of the miRNA candidates, we compare the candidates predicted by the intragenomic matching in each genome. We consider a candidate to be conserved if there exists a candidate in one of other genomes following the typical miRNA precursor conservation patterns [19,33]: (1) the mature miRNA sequences should be highly similar and should reside on the same arm of the precursor; (2) the loop region connecting the miRNA and miRNA* should be less conserved than both the miRNA and miRNA* (see Materials and Methods for details).

All candidate loci are compared and aggregated into families. We observe that the conserved miRNAs (including many miRBase miRNAs) are often members of multilocus families, while 35% of our predicted putative miRNAs are singletons. These loci may be of more recent evolutionary origin, not having undergone as many duplications as the deeply conserved miRNAs.

**Figure 5:**
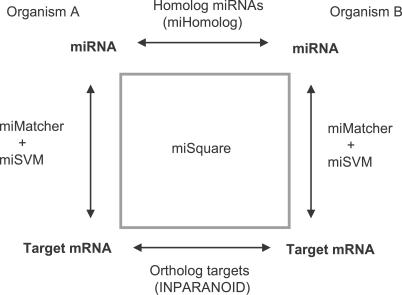
The Principle of the miSquare Conservation Criteria When two orthologous miRNAs have at least one instance of orthologous targets in the two organisms, we call this a miSquare.

Given that a miRNA candidate is conserved between two species, we investigate whether the conservation extends to a more functional level, namely if the two candidates have orthologous targets. When two orthologous miRNAs have at least one instance of orthologous targets in the two organisms, we call this a “miSquare” (Figure 5). For the purpose of identifying miSquares, we use an expanded target list based on looser matching criteria as detailed in Materials and Methods and [19].

We note that ∼90% of the candidates with a homolog in another species also share at least one target (putting them into the miSquare category), consistent with conservation of the regulatory function. Consistent with this, 60%–75% of the annotated miRBase miRNAs in each organism participates in at least one miSquare.

**Figure 6:**
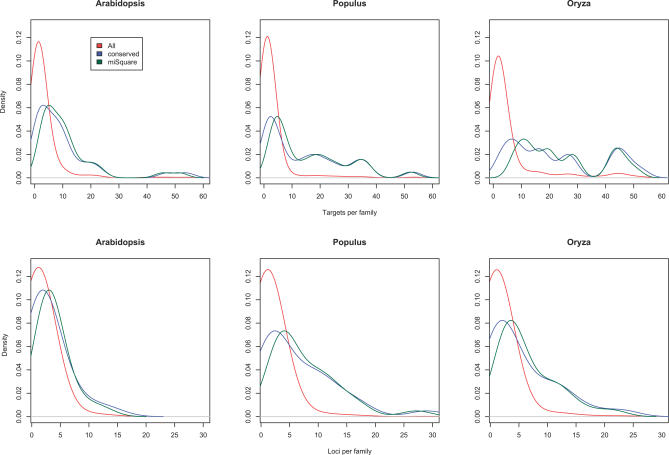
Distribution of Family Sizes and Target Numbers miRNA candidates outside coding sequences and repeat regions are counted and density plots constructed. Top row: Distribution of the number of targets per miRNA family. Bottom row: Distributions of family sizes. The conserved candidates generally have larger family sizes.

As can be seen in Figure 6, conserved miRNAs tend to have more targets than the nonconserved. This fact can be explained by the assertion that compensatory mutations between a miRNA and its target(s) are less likely to happen if the miRNA has many targets constraining its sequence.

**Figure 7:**
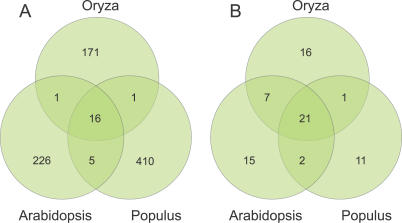
Conservation of miRNA Candidates and miRBase miRNAs (A) Species conservation (miHomology) of all candidate miRNA families predicted with miSVM and not overlapping repeat or coding sequence. The Venn diagram shows the number of families that are species specific and those that are conserved within another species (see Materials and Methods). (B) Species conservation (miHomology) of only miRBase (version 8.2) miRNA families (repeat/CDS overlapping families). We only include miRBase miRNAs that can be mapped exactly to the genome according to the reported precursor sequence and where we can predict at least one target.

Studying precursor conservation (miHomology) between the three species after filtering out candidates overlapping repeat and coding sequence, we find 226, 410, and 171 species-specific miRNA candidate families in *Arabidopsis*, *Populus,* and *Oryza,* respectively (Figure 7A). These families cover 272, 528, and 183 candidate miRNA loci in the three species. We find 16 miRNA families conserved in all three organisms. In *Arabidopsis,* all of these 16 conserved candidates are already annotated in miRBase, suggesting that most of the deeply conserved miRNAs are already found.

In an evolutionary perspective, one would expect more miRNAs to be common between the two dicots (*Arabidopsis* and *Populus*) than between a dicot and the monocot (*Oryza*). Our predictions are fully in agreement with this hypothesis: only a single family is conserved between pairs of *Oryza* and a dicot, while five families are conserved only between dicots. The picture is more ambiguous when we investigate all the miRNAs in miRBase and use the same family assignment criteria (Figure 7B). Most conserved miRBase miRNA families (21) are conserved between all three species. Unexpectedly, a high number of miRBase families (seven) are only conserved between the dicot *Arabidopsis* and the monocot *Oryza*: miR413, miR414, miR417–420, and miR426 (ath-MIR416 was not part of the analysis, as no targets could be predicted for this miRNA). These are miRNAs that do not pass the miMatcher pipeline and whose validity, as mentioned earlier, has been questioned in a recent review [32]. There are only one to two miRBase miRNA families conserved between *Populus* and one of the other two species. This could be due to the fact that only few studies have looked at conservation in *Populus,* and no studies have looked at conservation only between *Populus* and *Oryza*.

### Novel miSquare miRNA Candidates

Among the predicted miRNA candidates, the conserved ones classified as miSquare miRNAs are most likely to be actively used and have a phenotypic impact. The majority of predictions in this category are identical or overlapping with the already known miRBase miRNAs, because similar criteria have been used before to identify new miRNAs [5,19]. We did a manual assessment of the potential novel miSquare candidates that do not overlap other miRBase miRNA precursors or known annotated coding regions or repeats. In *Arabidopsis*, the two candidates are the miRNA* sequences of MIR172 precursors. Interestingly, Wang et al. have found Northern blot expression evidence of the ath-MIR172b* sequence [6]. In *Oryza* and *Populus,* we find no new miSquare families, but three new members of known miRBase families (oza-MIR399, ptc-MIR166, and ptc-MIR395; see Table 2).

**Table 2:**
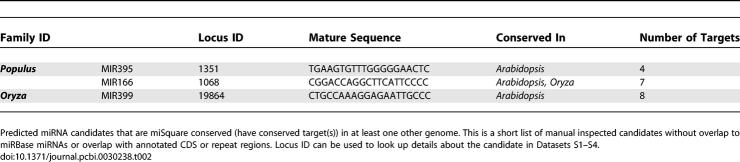
Novel miSquare Conserved miRNA Candidates

### Distribution of Candidates in the Genomic Landscape

Both miRBase miRNAs and our predictions are found in many different genomic contexts. Analyzing the genomic context of a miRNA can provide hints to its function.

**Figure 8:**
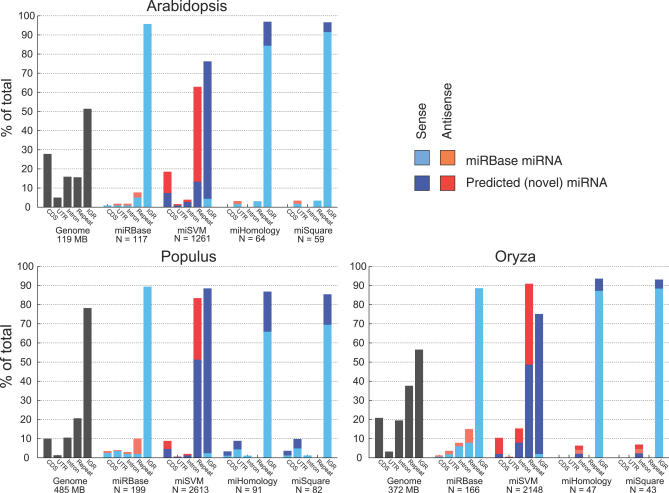
Distribution of miRNAs in the Genomic Landscape A histogram for each of the three organisms showing the genomic origin of the miRNAs. The first histogram group in each plot shows the relative abundance of coding (CDS), untranslated (UTR), intron, repeat, and intergenic (IGR) regions in the genome. The second histogram group shows the relative abundance of miRBase miRNAs among these regions, with different colors for sense and antisense overlap. The last three histogram groups capture the same measurements for predicted miSVM, miHomology, and miSquare miRNAs. Novel predicted miRNAs (not found in miRBase) in these groups are illustrated with darker colors, whereas miRBase miRNAs found among our candidates have lighter colors (see legend).

In contrast to animals (with ∼40% of miRBase human miRNA loci in introns), the three plants studied here have the vast majority of the miRBase miRNAs in intergenic regions (Figure 8). *Oryza* has the highest fraction (∼8%) of both miRBase miRNAs and predicted miRNA candidates derived from introns in sense direction.

miRBase miRNAs contained in protein-coding genes are clearly underrepresented relative to the fraction of the total genome. The conserved and miSquare subsets of our predictions show a similar underrepresentation, whereas the rest of the candidates have a larger fraction overlapping already annotated genes, although still underrepresented in intron and CDS regions compared to the total CDS/intron fraction of the genomes.

When on the same strand as another gene, the CDS-, untranslated region–, or intron-mapping candidates are interesting cases, since they could constitute parallel signals that are sent when the “host” is expressed. In contrast to “normal” sense–antisense pairs, supposedly forming dsRNA to trigger the RNAi machinery (reviewed in [34]), miRNAs encoded on the antisense strand to a protein-coding gene suggest an alternative and easily evolvable way of regulating the sense transcript.

### Function of Candidates

**Figure 9:**
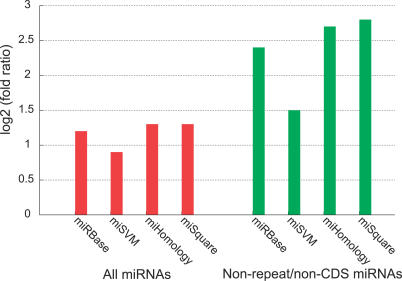
miRNA Candidates Targeting TFs in *Arabidopsis* Enrichment of *Arabidopsis* TF targets in different sets of miRNAs, comparing the relative abundance of TFs among the miRNA targets with the relative abundance of TFs in the *Arabidopsis* genome (∼5.9%). For the nonfiltered miRNA sets (red), the relative abundance of TF targets are miRBase, 59 of 440; miSVM, 87 of 782; miHomology, 60 of 429; and miSquare, 59 of 408. For the repeat/CDS filtered miRNA sets (green), the numbers are miRBase, 42 of 133; miSVM, 73 of 442; miHomology, 43 of 116; and miSquare, 42 of 103.

While the first reports of miRNA targets in plants found that a large proportion of the targets were transcription factors (TFs) [28], subsequent research has suggested that plant miRNA targets are more diverse although still enriched in TFs [6,17,18]. To test whether the targets for our miRNA candidates are enriched in TFs, we use the *Arabidopsis* TF database AtTFDB [35]. The enrichment is found as the fraction of predicted targets that are TFs divided by the fraction of all annotated genes that are TFs (5.8%). The results are shown in Figure 9 for different sets of miRNAs with and without repeat/CDS overlapping miRNAs: miRBase, miSVM, miHomology, and miSquare miRNAs.

All sets show a high enrichment of TF targets (miRBase, miHomology, and miSquare of almost identical magnitudes). When we filter out miRNAs that overlap repeat/CDS regions, the TF target enrichment rises notably for all sets, indicating a different functional profile of CDS/repeat-derived miRNA candidates. The enrichment tops for non-repeat/CDS miSquare miRNAs, with 40.8% (2.8-fold enrichment) of the targets being TFs. This high TF target enrichment of conserved miRNAs suggests that miRNA interaction with the core gene regulatory machinery is an important evolutionary feature.

Our candidates (miSVM) show a lesser but still considerable enrichment compared to miRBase and conserved miRNAs (both with and without repeat/CDS-overlapping miRNAs). This implies that a larger proportion of the nonconserved miRNA candidates have targets outside the core gene regulatory machinery. These observations suggest that a notable fraction of our nonconserved miRNA candidates are functionally different than the conserved miRNA candidates and already known miRNAs. This can be interpreted in at least two ways. It could be that the fraction of estimated false positives has targets spread uniformly throughout the genome and thereby lower the total enrichment of TF targets in our candidate set. On the other hand, it makes biological sense that newly evolved (or evolving) miRNAs arise uniformly around the genome with targets uniformly spread on all mRNAs, and only the functionally important ones then being maintained through evolution.

### Experimental Evidence for Nonconserved Candidates in *Arabidopsis*

Recently, deep sequencing of small RNAs in *Arabidopsis* using the 454 technology has revealed novel nonconserved miRNAs [36,37]. In one study [36], small RNAs (16–28 nt) were sequenced from libraries made from whole seedlings, rosette leaves, whole flowers, and siliques, resulting in approximately 340,000 unique sequences with a perfect match to the genome. Applying very strict filters including a requirement for expression of both the mature miRNA and miRNA*, the authors identified 38 high-confidence novel nonconserved miRNAs among the sequences.

**Figure 10:**
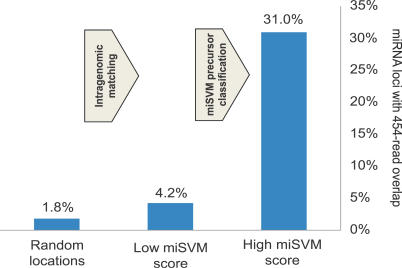
miRNA Overlap with Sequenced Small RNAs Percentage of *Arabidopsis* miRNAs with 20–23 nt coordinate overlap with sequenced and genome-mapped small RNAs from [36]. Three different sets are shown (all filtered for CDS/repeat overlap). (A) Random 22mers, 21.549 loci sampled randomly from the genome. (B) A set of 1,886 miRNA loci classified as non-miRNAs with miSVM. (C) A set of 334 miRNA loci classified as miRNAs by miSVM.

The full database of genome-mapped small RNAs from this sequencing study covers 5% of the *Arabidopsis* genome. A total of 31% (104) of our 334 candidates overlap with an observed small RNA with 20–23nt. Comparing this overlap frequency to (1) 22mers randomly chosen from the genome (1.8% overlap with 454 reads), and (2) miRNA candidates found by intragenomic matching but removed with miSVM (4.2% overlap) (both sets filtered for CDS/repeat overlap), it can be seen that both the intragenomic matching and miSVM step improves the frequency of miRNA candidates expressed by small RNAs (Figure 10).

Of the 104 miRNA candidates with read overlap, 74 are already in the new miRBase 9.1 (comprising 184 miRNA precursors, including the findings from Rajagopalan et al. [36] and Fahlgren et al. [37]). This leaves us with a short list of 28 novel nonconserved miRNA precursor candidates where the predicted mature miRNA has been observed experimentally (see Dataset S4).

### Conclusion

By using intragenomic matching in a single genome followed by hairpin classification, this work demonstrates that miRNA candidates can be found via their targets with high specificity and reasonable sensitivity. Using this approach, we have found surprisingly large numbers of miRNA candidates in the three plants studied.

While most of the miRBase miRNAs are conserved along with their targets in other plant species (although some newly discovered are more species specific, e.g., [38,39]), the majority of the candidates found by our approach seem to be specific for each genome. Many of our candidates have a different genomic origin than the known miRNAs: many are encoded in regions annotated as repeats or protein CDS (both sense and antisense).

Recently, it has been shown that repeat associated miRNAs are common in animals [22,26]. Similarily, in plants we find that a large fraction of the new miRNA candidates derive from repeat regions. This suggests an active role for repeats in the regulation of gene expression.

Their functional profile also differs from already known miRNAs in the sense that there is less target overrepresentation among TFs.

Recently, deep sequencing of small RNAs in *Arabidopsis* using the 454 technology has revealed many novel nonconserved miRNAs in *Arabidopsis* [36–38]. Of our 334 predicted *Arabidopsis* miRNA candidates outside repeat and protein annotation, we identify 28 novel candidates with experimental support from a small-RNA sequencing project (see Dataset S4).

Together, these observations raise some important questions: how many of the candidates are actually functional? Do these nonconversed miRNAs play a role in speciation? Conversely, if they are not functional, we must ask why: does something prevent their transcription or maturation? For example, in *Arabidopsis,* we know that many intergenic regions and regions antisense to annotated genes are transcribed [14]. If they are transcribed, what prevents a candidate miRNA from being functional? We know that their structure looks like that of known miRNAs and that they match at least one target with maximum two mismatches—just like the experimentally confirmed miRNAs. What other unknown features of sequence and structure, if any, are required for a miRNA-like hairpin to be functional? We hypothesize that the candidates that are not (yet) functional form a pool from which functional miRNAs can evolve in relatively few steps, thus facilitating adaptation towards new niches by improving the organisms' evolveability.

## Materials and Methods

### Input data.

*Known miRNAs.* Sequences were downloaded from miRBase release 8.2 [40]. A total of one *Populus* (ptc-MIR481a) and eight *Oryza* miRBase (osa-MIR444, osa-MIR445b/c/e/f/g/h/i) genes were discarded because their reported precursor sequences could not be mapped to the genome. This leaves us with 118 (*Arabidopsis*), 212 (*Populus*), and 174 (*Oryza*) genome-mapped miRNA genes. Requiring nonoverlapping genome loci and at least one predicted target, these numbers are further reduced to 117 (*Arabidopsis*), 199 (*Populus*), and 166 (*Oryza*) unique miRNA genes (see miMatcher procedure and grouping into loci explained below).

Arabidopsis thaliana genome and annotation TAIR assembly version 6 were downloaded from http://www.arabidopsis.org. We only use RefSeq protein-coding mRNAs as possible miRNA targets.

Populus trichocarpa genome assembly and annotation used was kindly provided by Eric Bonnét and is available upon request. The official release of the genome is now available at http://genome.jgi-psf.org/Poptr1_1.

*Oryza sativa.* TIGR assembly version 4.0 and annotation was downloaded from ftp://ftp.tigr.org.

### The miMatcher procedure.

This is an improved version of the procedure described in [18].

*Finding initial micromatches.* For each annotated spliced mRNA, we search the genome for matches of length at least 20 with a maximum of two mismatches (no gaps or wobbles allowed) using the suffix array–based program vmatch (http://www.vmatch.de). This is an exhaustive search guaranteed to find all matches.

*Prefiltering the intragenomic matches.* The initial micromatches are filtered by discarding all matches not fulfilling the following criteria.

*Attributes of the putative mature sequence.* Shannon index entropy of the genomic part of the match (putative mature miRNA sequence) must be larger than 1.7 bits. In addition, the following must hold: (1) all four bases had to be present at least once; and (2) at most, 11 of the three most frequent dinucleotides in the sequence were allowed. Length of the genomic part of the match must be 20–25 nt (both inclusive).

*Attributes of the intragenomic match.* Using the program RNAcofold (Vienna RNA package [41]), the free energy change when a miRNA candidate binds to a target site was calculated. The free energy of binding per base must be less than −1.4 kcal/mol.

*Attributes of the precursor structure.* In order to predict a possible precursor molecule, two genomic sequences around each micromatch are extracted: one starting 10 bases 5′ of the micromatch and extending 240 bases 3′ of the micromatch, and one with the extension lengths reversed. Each of these is treated independently in the following analysis. First, the potential precursor sequence is folded with RNAfold [41] to find the minimum free energy structure. The complementary part of the miRNA in this stem is denoted miRNA*, and is found as the sequence of nucleotides delimited by the pairing partners of the most 3′ and 5′ bases in the mature sequence. We define the attribute *pretty stem* to be true if all base pairs involving the mature microRNA and miRNA* are pairing to bases in the same direction opposite to each other.

*Trimming the precursor*. Since all pre-miRNA are not of the same length, we trim down the initially found constant length pre-miRNA structure. We count how far inward toward the loop or outward toward the ends of the RNA sequence the stem extends using the following algorithm: moving out from the terminal base pair between the miRNA and miRNA*, a score of 1 is assigned for each base pair encountered and a score of −1 for each unpaired base. The extension is stopped when the current score is less than 5 lower than the maximum score so far. The last base pair is considered the terminus of the trimmed precursor.

Given the predicted minimum free energy secondary structure of the putative miRNA precursor, we calculate the following attributes: pairs to mature miRNA—the number of paired bases in part of the precursor predicted to become the mature miRNA; outer and inner extension—found during the trimming procedure described above; distance between miRNA and miRNA*—the number of nucleotides between the bases participating in the innermost base pair of the mature miRNA; stability of precursor: this is simply calculated by using RNAfold on the trimmed precursor and dividing by the number of bases. This is based on the observation that miRNA precursors are unusually stable [42]; asymmetrically unpaired bases in stem—we count unpaired bases in either the miRNA or miRNA* where there are no corresponding unpaired base on the other side; and 5′ and 3′ stem hybridization—the energy gain calculated by RNAcofold (Vienna RNA package) from hybridizing the ten first or last bases of the mature miRNA to miRNA*.

It should be noted that the structural attributes are not necessarily strictly independent from each other (e.g., a long “inner extension” correlates with the “distance between the miRNA and miRNA*”).

### miSVM: SVM training.

We used SVM software implemented in the *SVMlight* package (downloadable from http://svmlight.joachims.org) using a radial kernel and double penalization of errors on the (smaller) set of positive examples. The input to the SVM is the structural features detailed above.

*Cross-validation.* To avoid overtraining and to get a realistic evaluation of the ability of the SVM to generalize, it is important to reduce redundancy between training and test sets. Because precursors in the same family often have similar structures, we performed “leave-one-family-out” cross-validation to assess generalization across families. The positive examples (miRBase miRNAs) were divided into families according to homology (we used the families provided by miRBase). For each family, a training set was constructed from the remaining positive examples, and all but 100 of the negative examples were chosen by random. The SVM was trained on this training set and subsequently tested on the withheld family and negative examples. The final SVM was retrained on the entire dataset and is called miSVM.

### Grouping of candidates into genomic loci.

Given the location (coordinates and strand) of the mature part of a miRNA precursor, we assign miRNA candidates into genomic loci by grouping precursors with up to 4 nt overlap of the mature sequence together. In *Populus,* the 212 miRBase 8.2 genome-mapped genes correspond to 200 unique genomic loci; in *Oryza,* the 174 miRBase genes are reduced to 167 loci. All 118 *Arabidopsis* miRNAs are correctly mapped to unique loci.

### Position in the genome relative to existing annotation.

Gene models provided by the genome sequencing and annotation groups were downloaded (see above for sources), parsed, and read into database tables indexed by the absolute genomic coordinates. RepeatMasker (http://www.repeatmasker.org) was run to identify repeats whose locations were also stored in the database. In addition, we consider a candidate a repeat if it has a copy number (number of exact genome matches with length 20 allowing two mismatches or indels—corresponding to our miRNA family definition) greater than 100. In *Arabidopsis*, this copy number constraint annotates three miRBase miRNAs (ath-MIR415, ath-MIR401, and ath-MIR414) as repeats, two of which were already assigned as repeats by RepeatMasker. Similarly for *Oryza*, 16 miRBase miRNAs are annotated as repeats (15 were already assigned by RepeatMasker), and for *Populus*, 20 miRBase miRNAs are annotated as repeats (17 were already assigned by RepeatMasker).

All candidates where checked against this database to locate overlaps with annotation. When we consider the nonrepeat/CDS overlapping miRNAs, we remove miRNAs overlapping repeat or CDS regions (regardless of strand).

### Grouping of candidates into families.

All candidate miRNAs were grouped into families on the basis of mature sequence similarity: two candidates were grouped together if they shared at least 20 nucleotides allowing two mismatches or indels. Larger family clusters were constructed using single linkage clustering. In addition, it is required that all members of a family must have the mature miRNA on the same arm of the precursor. These criteria gave us near-perfect recovery of the miRBase-assigned families (miRBase version 8.2). In *Arabidopsis*, only the miR171 family is divided in two families, and the following miRBase families are pairwise grouped together: MIR319–MIR159, MIR156–MIR157, MIR165–MIR166, and MIR170–MIR171.

### Determination of homology between miRNAs in different species—miHomology.

To determine if two miRNA precursors from different species are homologous, we require fulfillment of two criteria: (1) the mature miRNAs must align over a region of minimum 20 bases with a maximum of two mismatches (gaps count as mismatches), and be on the same arm of the precursor; and (2) no 20mer in the loop region (connecting the miRNA and miRNA*) may align better than the miRNA or miRNA* region. We explored the effect of these criteria on a few expected positive and negative miRNA test cases. As a positive case, we classify the three miR172a miRNAs from *Arabidopsis*, *Oryza*, and *Populus* as homologs (the same is true for miR156a—no other similar cases were explored). Testing the *Arabidopsis* ath-MIR169 family (14 members), approximately two-thirds could be grouped as homologs: this is as expected, as precursors originating from recent duplications have highly similar loop regions [33]. As a negative test case, we took 21 *Arabidopsis* “a” precursors (ath-MIR156a, ath-MIR157a, etc.) and found only two homologous pairs based on our test: ath-MIR156a–157a and ath-MIR165a–166a. These two pairs are often considered to be from the same miRNA families.

We consider two miRNA families from different species as conserved if there exists a precursor in each family with homology (miHomology) to a precursor in the other family. Because miRNA families are computationally determined in a genome-dependent manner (relying on single linkage clustering), there can be a minor asymmetry in miRNA family conservation: looking from *Arabidopsis,* there can be *X* families conserved in *Populus*, while looking from *Populus,* there can be *Y* families conserved in *Arabidopsis*. In this paper, we report the larger of these two numbers as the family conservation count.

### Finding and scoring conserved miRNA targets—miSquare.

To identify conserved regulatory interactions between a miRNA and target in different species—miSquares—we have two tasks: (1) determine protein orthology between the species, and (2) determine the targets of the conserved miRNAs.

Protein orthology in the three organisms was determined using the INPARANOID program [43]. The program uses bidirectional best BLAST hits to determine orthologs between two species. In addition, it BLASTs each proteome against itself to determine “inparalogs”—presumed gene duplications after speciation. The program was run using Caenorhabditis elegans (wormpep157 from Wormbase) as outgroup, and otherwise default parameters.

The intragenomic matching procedure simultaneously finds miRNAs and corresponding targets with up to two mismatches (no wobbles or gaps allowed). According to Jones-Rhoades and Bartel [19], we can find targets above noise with a weaker matching criterion if we add target homology as a constraint. With the exception that we count wobbles as mismatches, we use the same matching and scoring rules as presented in this paper. Given a miRNA, we find target sequences that align over 20 nucleotides with a score ≤3 according to the scoring scheme: mismatch scores as 1, gap (open and extension) scores as 2. The original article argues for a cutoff score of 3.5 because they score wobbles less restrictively (score .5). In other words, our scoring scheme allows for targets with up to three mismatches or a combination of one gap and one mismatch. Based on these target requirements, we cannot find any targets for three miRBase 8.2 miRNA genes: ath-MIR416, ptc-MIR482, and osa-MIR438.

It should be noted that the miSquare criterion does not require the miRNAs in the two species to target homologous regions in the orthologous target mRNAs. We note, however, that in reality, this is most often the case.

### Experimental evidence for miRNA candidates.

We used the full database of sequenced genome-mapped small RNAs from the supplementary data of [36]. Our miRNA candidates were analyzed for overlap with these sequenced small RNAs by requiring a 20–23 nt coordinate overlap with the mature sequence of a candidate.

## Supporting Information

Dataset S1 Predicted miRNA Candidates in *Arabidopsis*
(606 KB TDS)

Dataset S2 Predicted miRNA Candidates in *Oryza*
(2.0 MB TDS)

Dataset S3 Predicted miRNA Candidates in *Populus*
(981 KB TDS)

Dataset S4 Predicted miRNA Candidates in *Arabidopsis* with Experimental Evidence (1 KB TDS)

Table S1 Description of Datasets S1–S4
(33 KB DOC)

## Abbreviations

CDS: coding sequence
miRNA: microRNA
SVM: support vector machine
TF: transcription factor
