# Supplementary material for: Intragenomic Matching Reveals a Huge Potential for miRNA-Mediated Regulation in Plants
Source: PLoS Comput Biol. 2007 Nov 30;3(11):e238. doi: 10.1371/journal.pcbi.0030238 (PMC2098865; doi:10.1371/journal.pcbi.0030238)
Supplement: Table S1 — (33 KB DOC) [file pcbi.0030238.st001.doc]

# Description of additional files 1-4

The files describing the miRNA candidates (Additional file 1-3) include the following details:

| Locus_id | Internally assigned ID for the candidate miRNA genomic locus (see article methods for details). |
| --- | --- |
| Family_id | Internally assigned ID for the candidate miRNA precursor family (see article methods for details). |
| miRBase_id | miRBase ID is reported if the candidate miRNA locus overlaps a known miRNA from miRBase. |
| Location | Genomic accession, strand and coordinates of the candidate miRNA locus. |
| pre-miRNA | Predicted precursor sequence of the miRNA locus. (see article methods for details). |
| mature miRNA | Predicted mature sequence of the miRNA locus. |
| svm_score | SVM score assigned to the miRNA locus based on the precursor structure (see figure 3 and article methods for details). |
| JRCriteria | True/false variable denoting whether the precursor structure fulfills the plant specific precursor criteria proposed by Jones-Rhoades et al. (2006). |
| Genome context | The genomic context (cds, intron, utr, repeat etc.) of the miRNA locus. |
| miSquare with | The organism names are reported if the miRNA locus is conserved as a miSquare in other organisms. |
| Targets | The predicted targets of the miRNA locus. |
| Copy Number | Genomic copy number of the miRNA locus (see article methods for details). |

Additional file 4 contains the miRNA locus_id`s of novel Arabidopsis miRNA candidates overlapping expressed small RNAs from the Rajagopalan et al. (2006) 454 deep sequencing study.
